# Supplementary material for: Global distribution and decline of mangrove coastal protection extends far beyond area loss
Source: Nat Commun. 2024 Nov 26;15:10267. doi: 10.1038/s41467-024-54349-0 (PMC11599879; doi:10.1038/s41467-024-54349-0)
Supplement: Supplementary file 1 — Supplementary Information [file 41467_2024_54349_MOESM1_ESM.pdf]

Supplementary information for:

Global distribution and decline of mangrove coastal protection extends far beyond area loss

Supplementary Discussion and Supplementary Figures:

Height Change Trend

Originally, we wanted to demonstrate that mangrove protection capacity was previously inadequately characterized as area of mangrove, approximated as width of mangrove in transects used in this study. Implicit in this area model is that protection capacity will be experienced primarily as a reduction in area. Our results imply that this is not the case and that "average-height", acting as a surrogate for tree-density and patchiness changes, is principally responsible for MCPI changes from 2007 to 2019. Our results go much further and suggest that destruction (by whatever means: human and/or environmental) intensified along ocean boundary currents and dramatically reduced average-height. Of greater worry is that average-height is a surrogate for biomass that varies as $\sim H^{2.55}$; hence biomass will be decaying at incredible rates between squared-to-cubed of height decay.

The almost-universality of height destruction from climate change is expected as mangroves are "edge-dwellers" thriving at the land-sea interface which has been stable over millennia; now being "rapidly" flooded by sea-level rise, compounded by pressure from coastal settlements. If predominantly from static and dynamic sea-level rise, mangrove destruction must have been going on since sea-levels began to rise decades ago. Indeed, we should be able to model this as an exponential decay process on the assumption that mangroves are highly rooted to the land-sea interface and cannot recover as quickly as they are being destroyed:

$$\frac{dH}{dt} = -\alpha H \qquad (1)$$

Where H is mangrove height; α is the exponential decay parameter, which implicitly incorporates all effects such as climate change (predominantly), perhaps weak regrowth, and human destruction; and t is time in years. Ultimately, the validity of this model will be tested statistically against observation.

To test this model, we implicitly assumed for theoretical modelling purposes that the observed 2005 to 2019 changes are from destruction. We computed the decay parameter α using median height (or the most prevalent height) to model the exponential decay depicted by equation (1). Variations of these parameters by Clusters in 2007, shown in Supplementary Fig. 1, suggest these parameters are approximately constant except for Cluster 8 (very low decay rate) and Cluster 2 (fast: ~double mean decay rate). Excluding these Clusters, height decayed at about 4.85% per year, and Biomass at 13.74% per year. Cluster 8 is already of low height, and decay was a low 0.30% per year. Cluster 2, which had highest heights, decayed at more-than double the mean rate, equating to 10.4% per year. Mangrove heights of near constant decay were in the range of 3.9 to 13.6 m. Below this range, decay is reduced, whilst it is accelerated above this. For reference, the timescale for

height decay across the near-constant decay rate, from heights of 13.6 m to 3.9 m, is about 25 years. The fact that Cluster 8 grew so remarkably from 2007 to 2019, suggests that mangrove height destruction was already well-advanced.

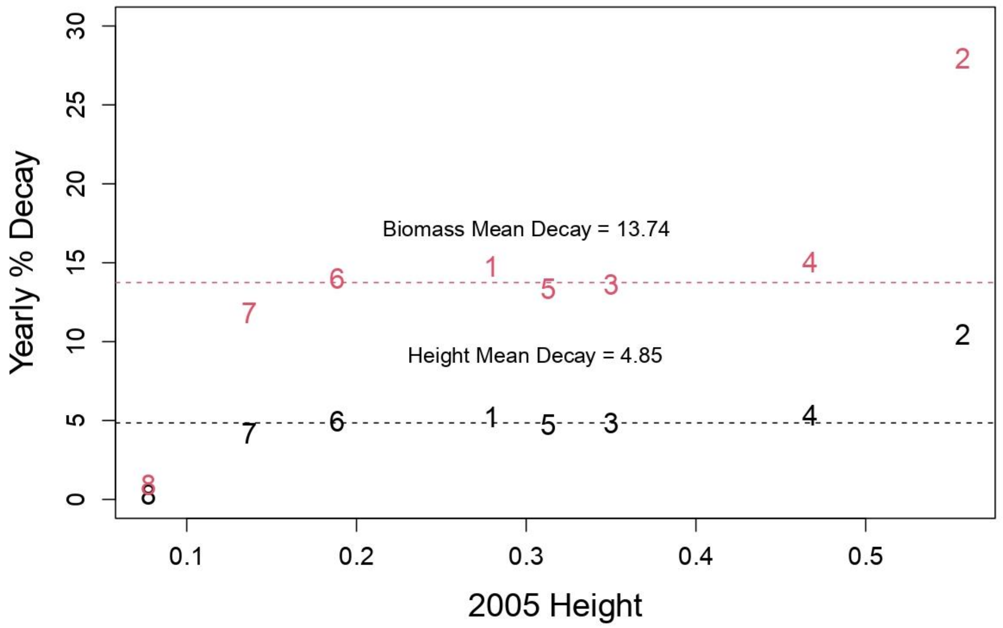

Supplementary Fig. 1 | Plots of the yearly % decay parameters (y-axis) for height (black numbers) and biomass (red numbers) for 2007-Clusters (numbers), and for 2005 height (x-axis).

The additional finding that significant height changes appear to coincide with oceanic boundary current regions could possibly imply that climate change forces from static sea-level rise plus dynamic height and kinetic energy increases may be acting in concert in these regions. Once started, relentless exponential destruction of mangroves is assured, because even at their very best state they can no longer withstand the intensified forces, so continuing destruction is assured. It is then a matter of how fast the destruction progresses.

The realization that height destruction was well in progress by 2007 raises the question of when mangroves were starting to be impacted (what year) and whether it was by climate change. Equation (1) could be used with the decay factors to determine heights in the past, assuming the decay factor doesn't vary. The low decay factor for Cluster 8 from advanced destruction implies this assumption is not correct and that we may have to use a factor that depends on height. To investigate a variable-decay model, we examined the decay by height. After some experimentation, the most reasonable and intuitive model was for mangroves less than 10 m height, a local positive relative decay rate of under 0.1/year, and a quadratic relationship between the decay factor (α) and height of the form:

$$\alpha = \theta * H (H0 - H) \quad (2)$$

Where, for small values of H, the decay is near-linear with H, whereas at $H = H0$, the “pristine” height, there is no decay and mangroves can fully protect the coast against prevailing forces. The fit of this model against observed exponential decay (from equation 1) and height in 2005, shown in Supplementary Fig. 2, was statistically highly significant with a reasonable adjusted-R of 0.75. For reference, including an intercept drops the adjusted-R to a low 0.015, despite significant parameters. Hence, the selected model is significant and reasonable statistically (and conceptually) with the following fitted parameters:

$$\alpha = 0.0156 * H - 0.00116 * H^2 \quad (3)$$

Defining features of this model are:

1. “Pristine” height: The statistically fitted model suggests $H0 = (0.0156/0.00116) = 13.45$ m, is the height at which full protection was achieved. Of course, this is a general statistical global model and may not apply locally, but it does suggest global destruction of mangroves under this height was taking place. This is larger than the 10 m we assumed in our biomass model, hence the height factor in MCPI may need to be modified as our current formulation is an underestimate. But it does suggest that “heights” in 2019 (median: 4.5 m) are quite low relative to heights (and more importantly biomass) required for full protection.
2. Maximum Decay α: This occurs where the derivative of equation (3) equals zero at $H = H0/2$. Hence, maximum decay rate (~0.052 /yr) is at $H_m = 6.7$ m, at half the pristine height as seen in Supplementary Fig. 2.
3. Maximum Destruction: Maximum height destruction occurs when the derivative of αH is zero, as shown in Supplementary Fig. 3. This occurs at $H_{md} = (2/3) *H0 = \sim 8.9$ m. This rate is $\alpha(H_{md}) * H_{md} = \sim 0.42$ m/yr.

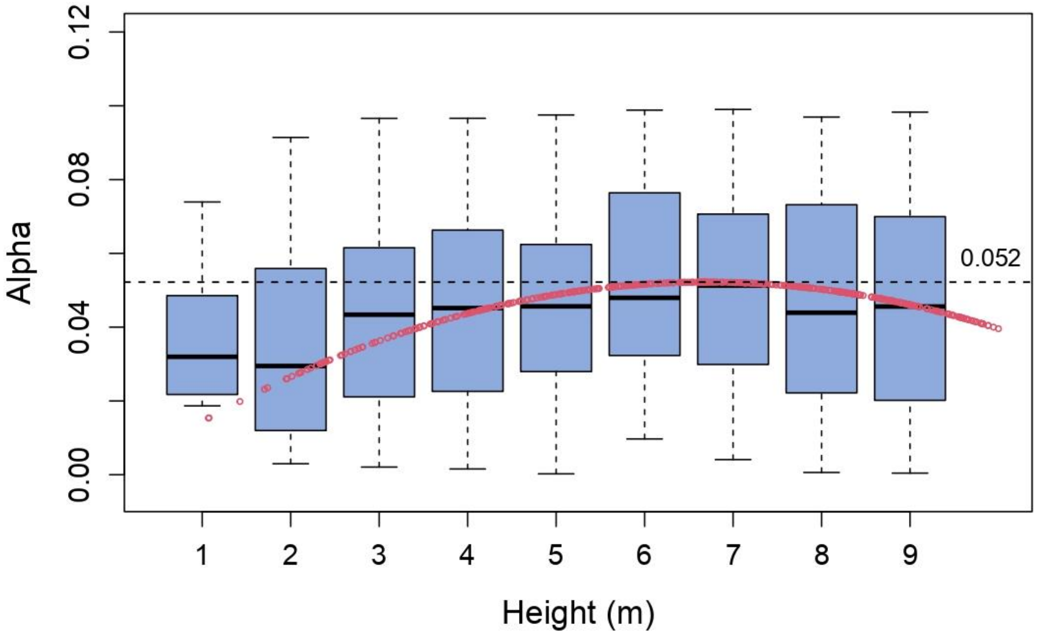

Supplementary Fig. 2 | Variation of height-decay factor (*α*) with height using boxplots (in blue) and a non-linear quadratic model (in red). Horizontal dashed-line is at the maximum decay of the quadratic model fit.

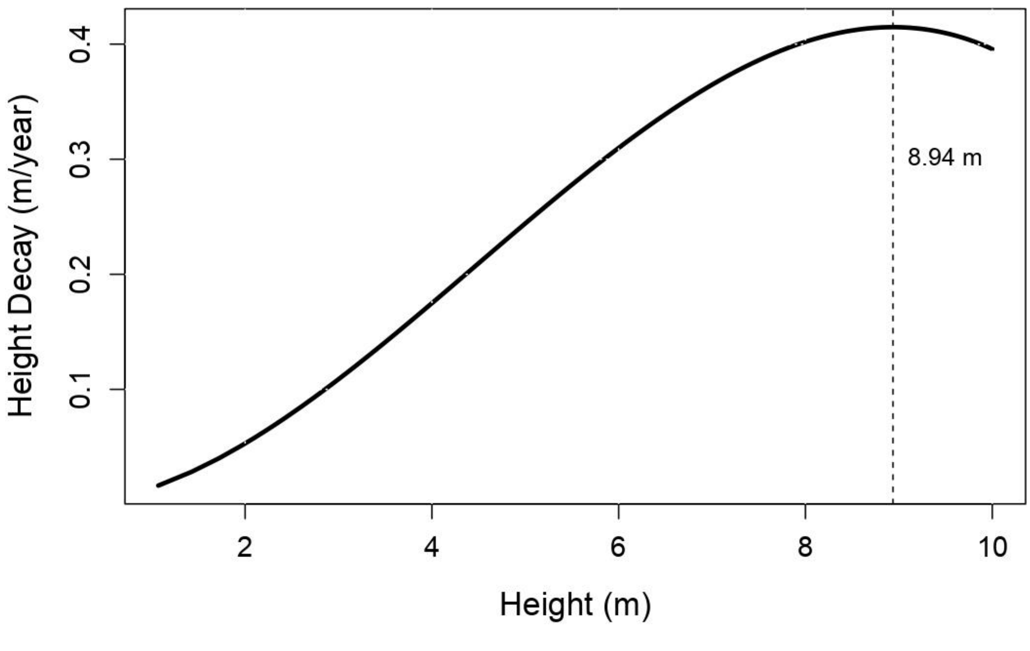

Supplementary Fig. 3 | Variation by height of yearly decay of height derived from the statistical model fit. The vertical dashed-line marks the height at which height destruction is maximal (two-thirds pristine height) at ~0.42 (m/year).

Our model clarifies that maximum height destruction rate (*αH*) occurs at a higher height than maximum

relative destruction rate (α). This height is quite tall at 8.9 m, as is the pristine height at 13.4 m. These values seriously challenge our empirical notions that mangroves more than 10 m tall provide excellent protection, as the model counters that it is in fact near-maximal destruction at that height.

The next step is to use those decay factors to determine when destruction began. Applying equations (1 and 3) backwards leads to the beginning (assumed pristine, or baseline) height *H0*, as:

$$H0 = H_{2019} \int_{2019}^{T_\infty} \alpha(H) H \, dt = H_{2005} \int_{2005}^{T_\infty} \alpha(H) H \, dt \tag{4}$$

Where, *H0* is the pristine height, *α(H)* is the height-dependent decay parameter from equation (3), H_{2019} (H_{2005}) is the median height in 2019 (2005), and T_∞ is the number of years from an estimated start of height decay till 2019 (2005). Theoretically, *H0* is never reached so a reasonable time needs to be assigned by looking at the approach to *H0* (say 99% *H0*) as shown by the reconstructed decay curves in Supplementary Fig. 4. Median global values of height in 2019 and 2005 suggest global destruction of mangroves began before 1989. This concords with suggestions by Kelly et al.[1] of visible signs of intensification of the East Australian Current in the 1990's. Note we could have started the backward integration from the height in 2005 (~8 m curve in Supplementary Fig. 4, with appropriate changes to x-axis) to end up with the same start time—simply because the exact same backward trajectory is being followed as it passes through 2005 from 2019.

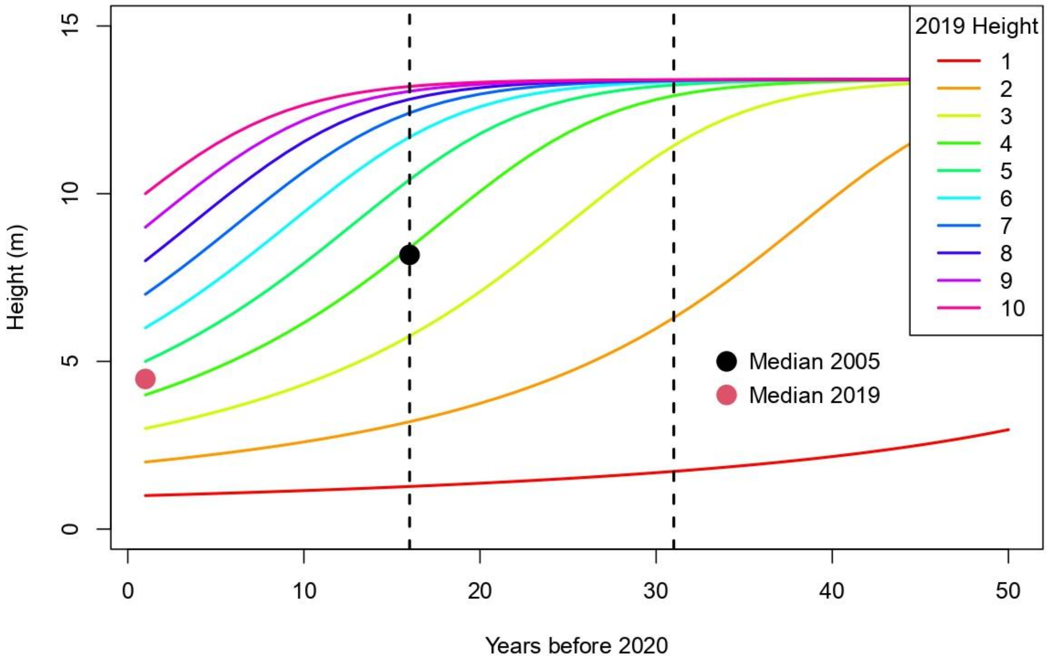

Supplementary Fig. 4 | Backward in time projection of mangrove average-height curves from an assumed start height in 2019, as shown in the Legend. The median global values from our data are shown by the red dot for 2019 (4.5 m) and by the black dot for 2005 (8.2 m). The approximate date at which destruction was in

progress of (2020 – 31) = 1989 is shown by the black vertical dashed-line to the right.

Trends for Width and NDVI (not normalized) do not lend themselves to similar modelling. For Width, decay is universally constant and low at 1.52% over the 12 years (adjusted-R^2 = 0.997). This universality may reflect emerging universal visual evidence of sea-level encroachment in remote-sensing images. In any case, this effect is "small" for now. For NDVI, a 2.34% increase is observed between the years (adjusted-R^2 = 0.988), but interestingly, including an intercept suggests an increase of 0.132 NDVI and a 19% decrease across the years; the fit is poorer with an adjusted-R^2 of 0.826. This decrease reflects the change of height from 2005 to 2019, real and/or statistical, combined with a larger increase in greening expected with the global rise in CO_2. For reference, height change (decrease) is 29% (over 14 years).

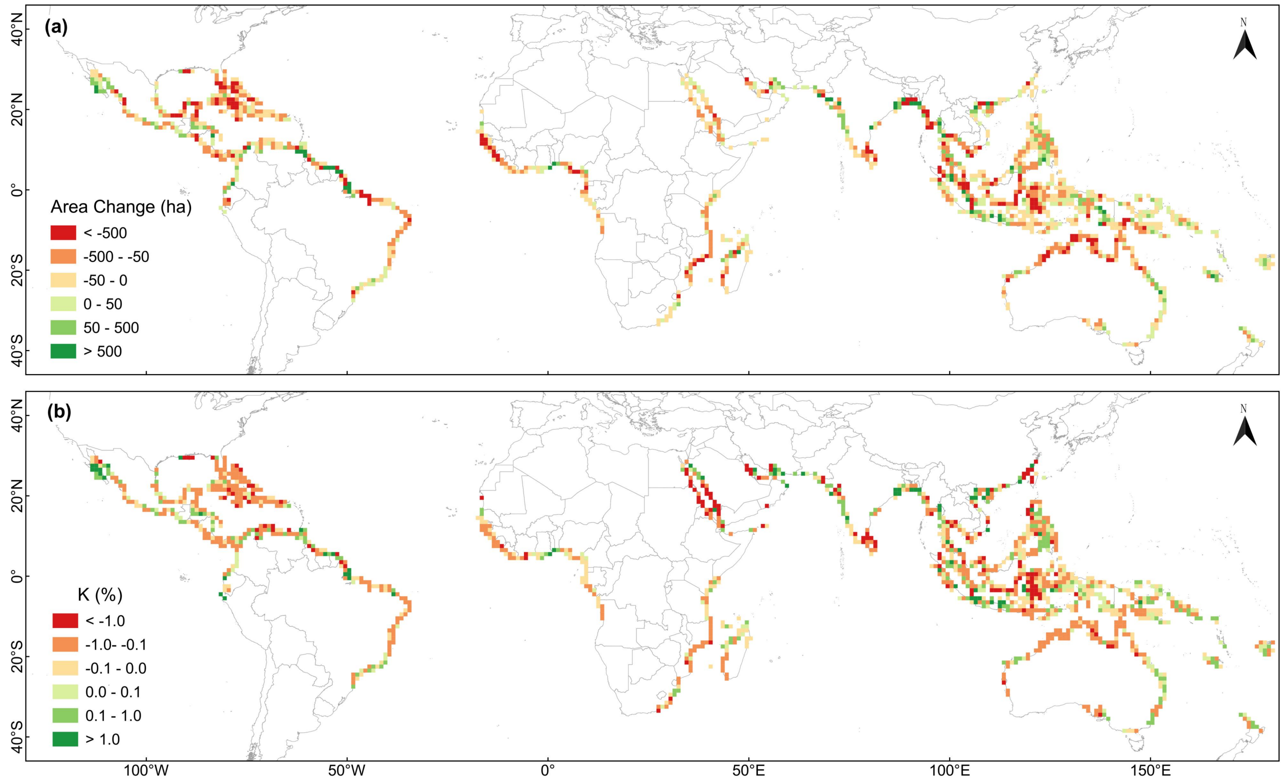

Supplementary Fig. 5 | Spatial variations in mangrove area changes based on the 2007 and 2019 mangrove distribution data. (a), (b) represent spatial distribution of change values and annual change rates respectively.

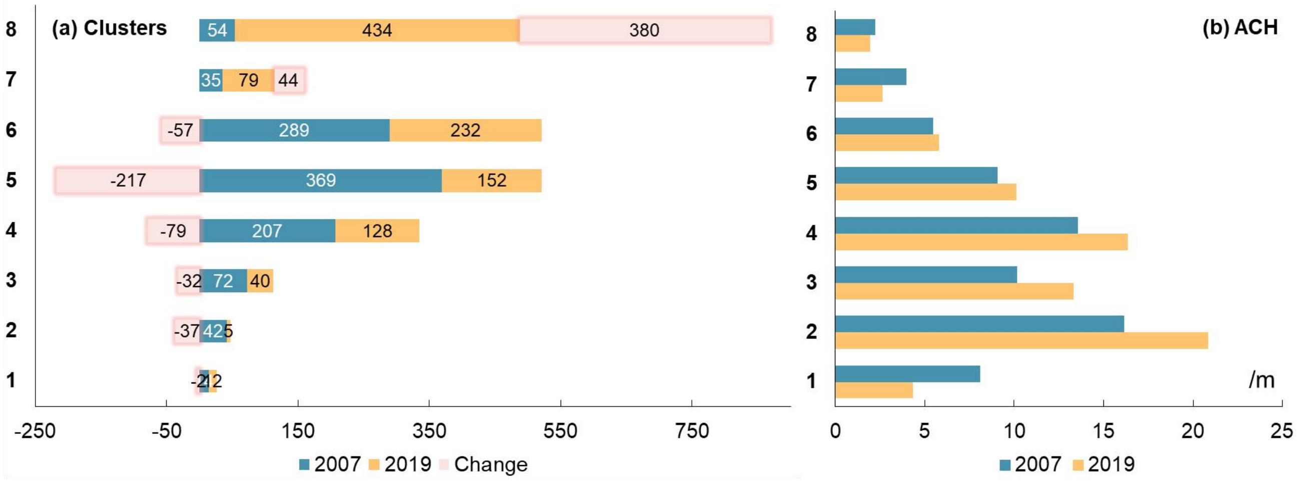

Supplementary Fig. 6 | (a) Bars showing number of cells in 2007 (blue), 2019 (orange), and the growth of cells (pink) as the difference between cells in 2019 and 2007. (b) Bars showing the ACH (Average Canopy Height) index for the Clusters for reference.

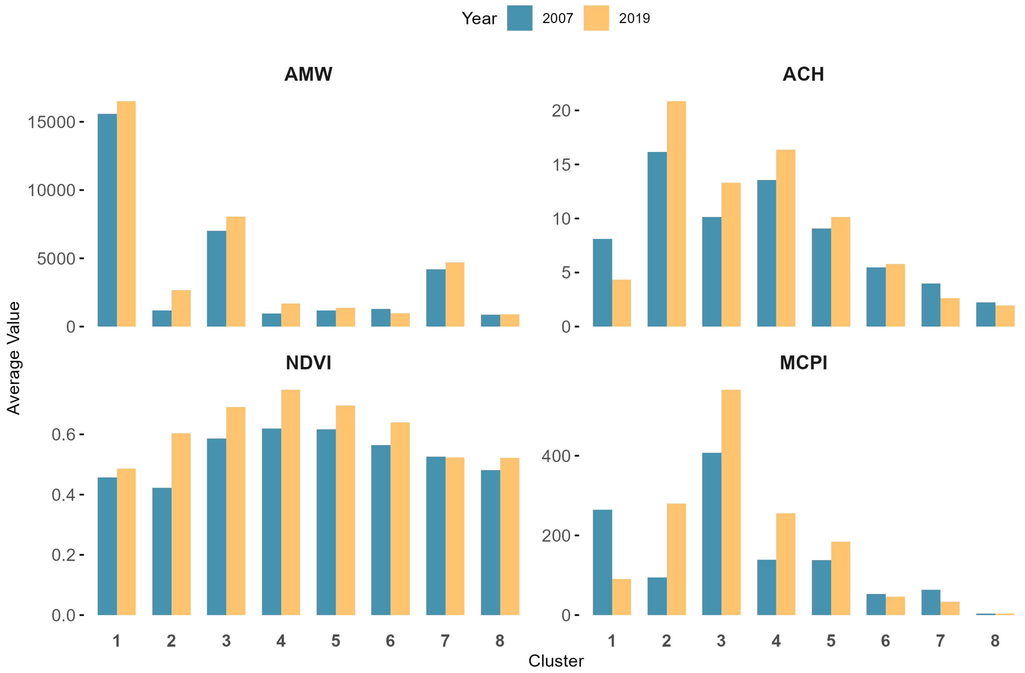

Supplementary Fig. 7 | Variation of factors used to construct MCPI (Mangrove Coastal Protection Index, see in Methods) and MCPI by Cluster.

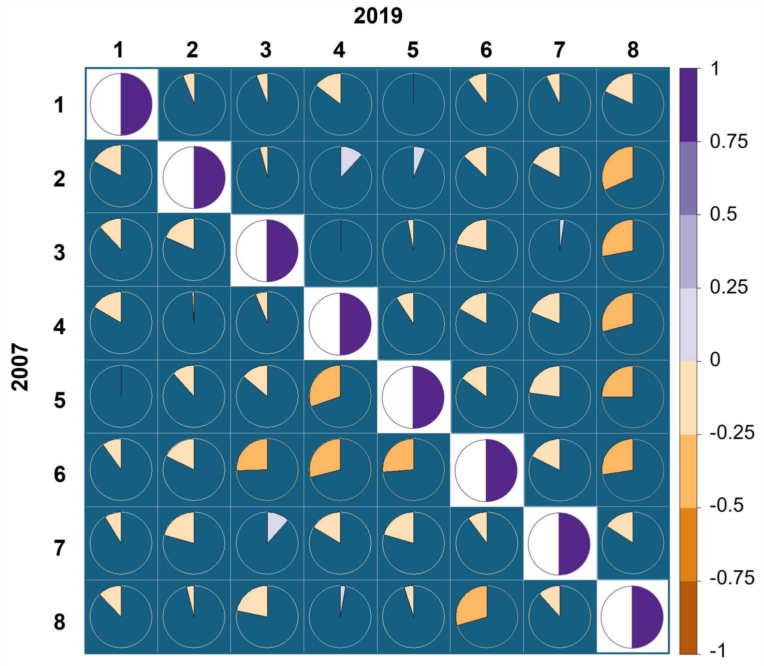

Supplementary Fig. 8 | Matrix showing correlations from the Cluster membership probabilities for 2007 and 2019 from Hopach's bootstrap estimate. Positive correlations are to the right and negative to the left, with diagonals = 1.

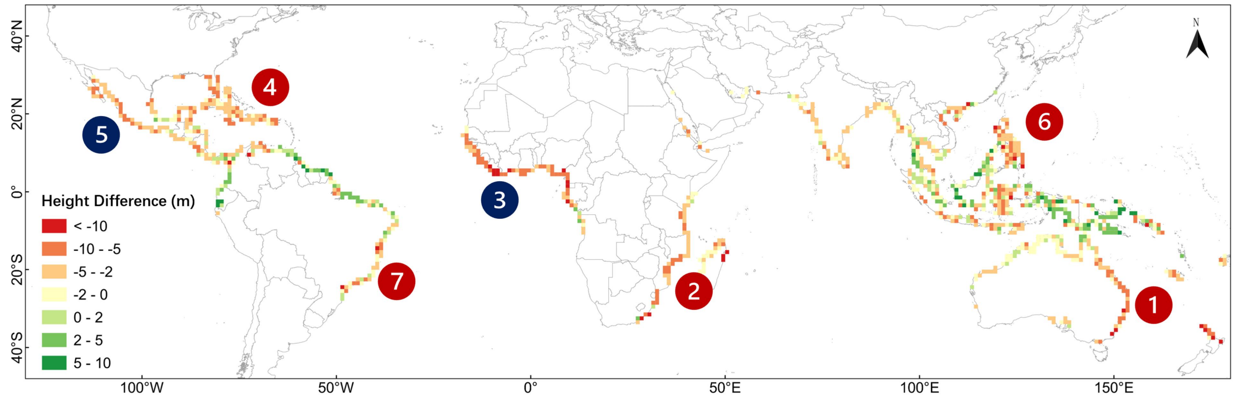

Supplementary Fig. 9 | Difference of height factor between 2007 and 2019. Note large height reductions in numbered boundary current regions of: (1) East Australian Current; (2) Agulhas Current; (3) Equatorial/Benguela Current; (4) Start of Gulf Stream Current; (5) California Current; (6) Kuroshio Current; and (7) Brazil Current. "Warm" currents (low to high latitude) are in dark-red, and "cold" ones (high to low latitude) in dark-blue.

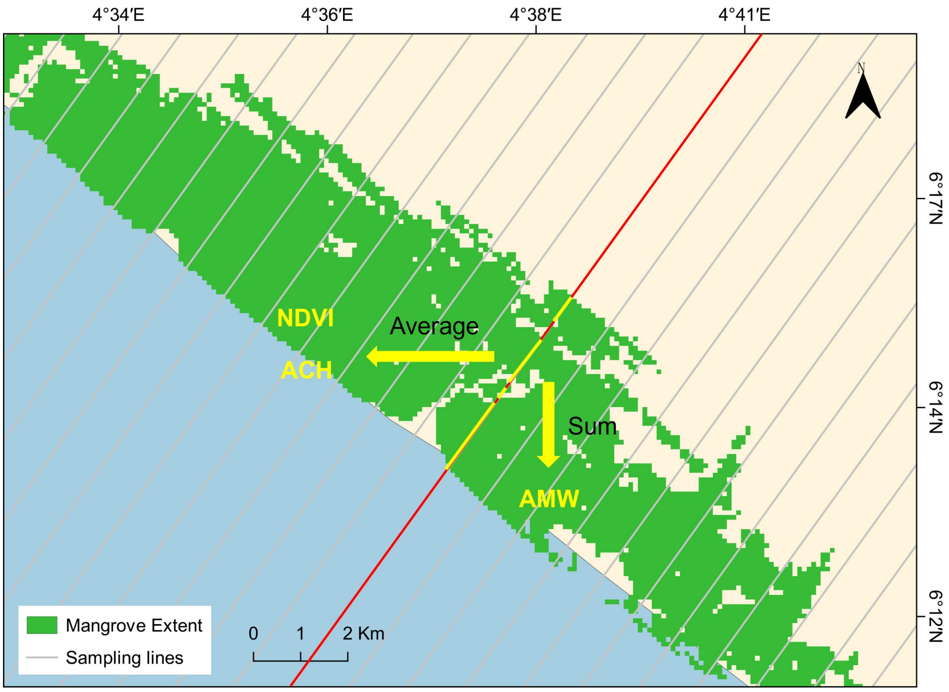

Supplementary Fig. 10 | Illustration of the method for extracting indicators using sampling lines.

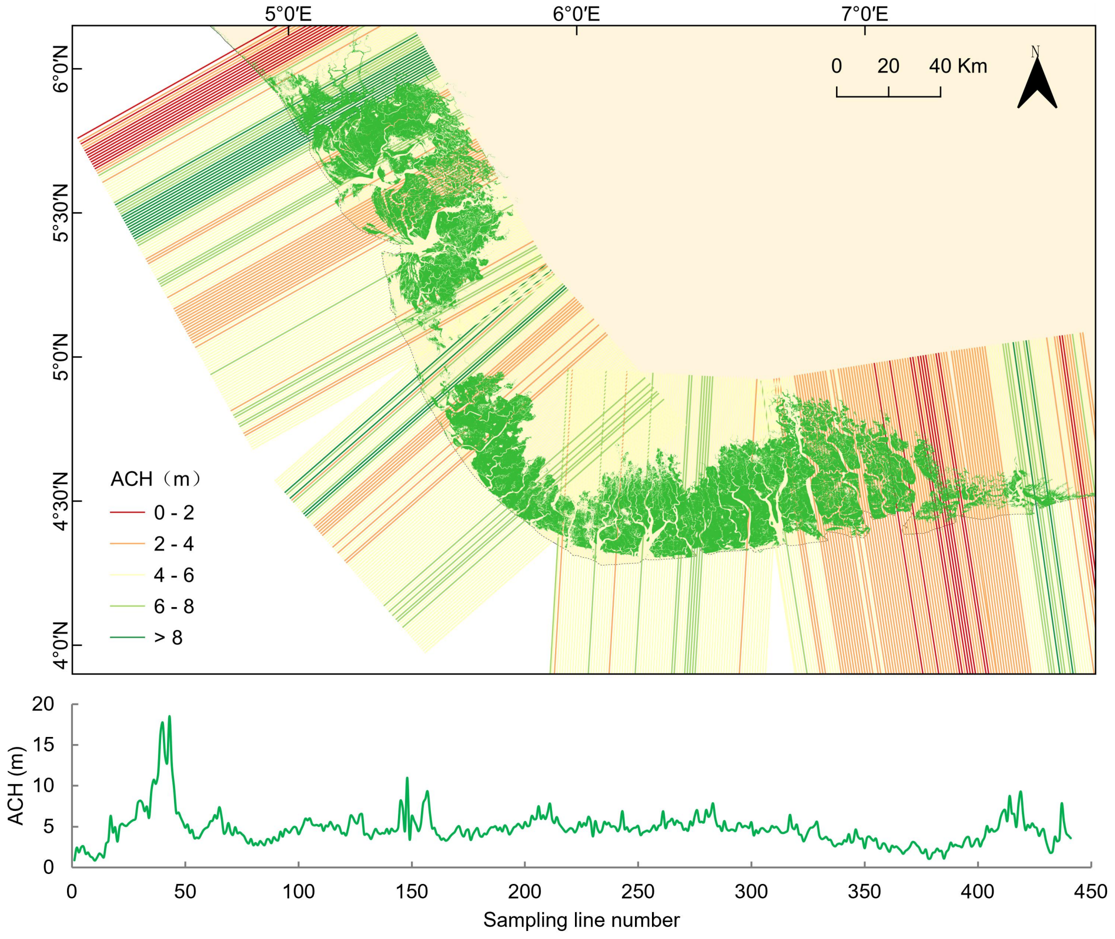

Supplementary Fig. 11 | Extraction of Average Canopy Height (ACH) results. Taking mangroves near the Gulf of Guinea as an example, sampling lines were generated perpendicular to the coastline, and average canopy height was extracted for each sampling line.

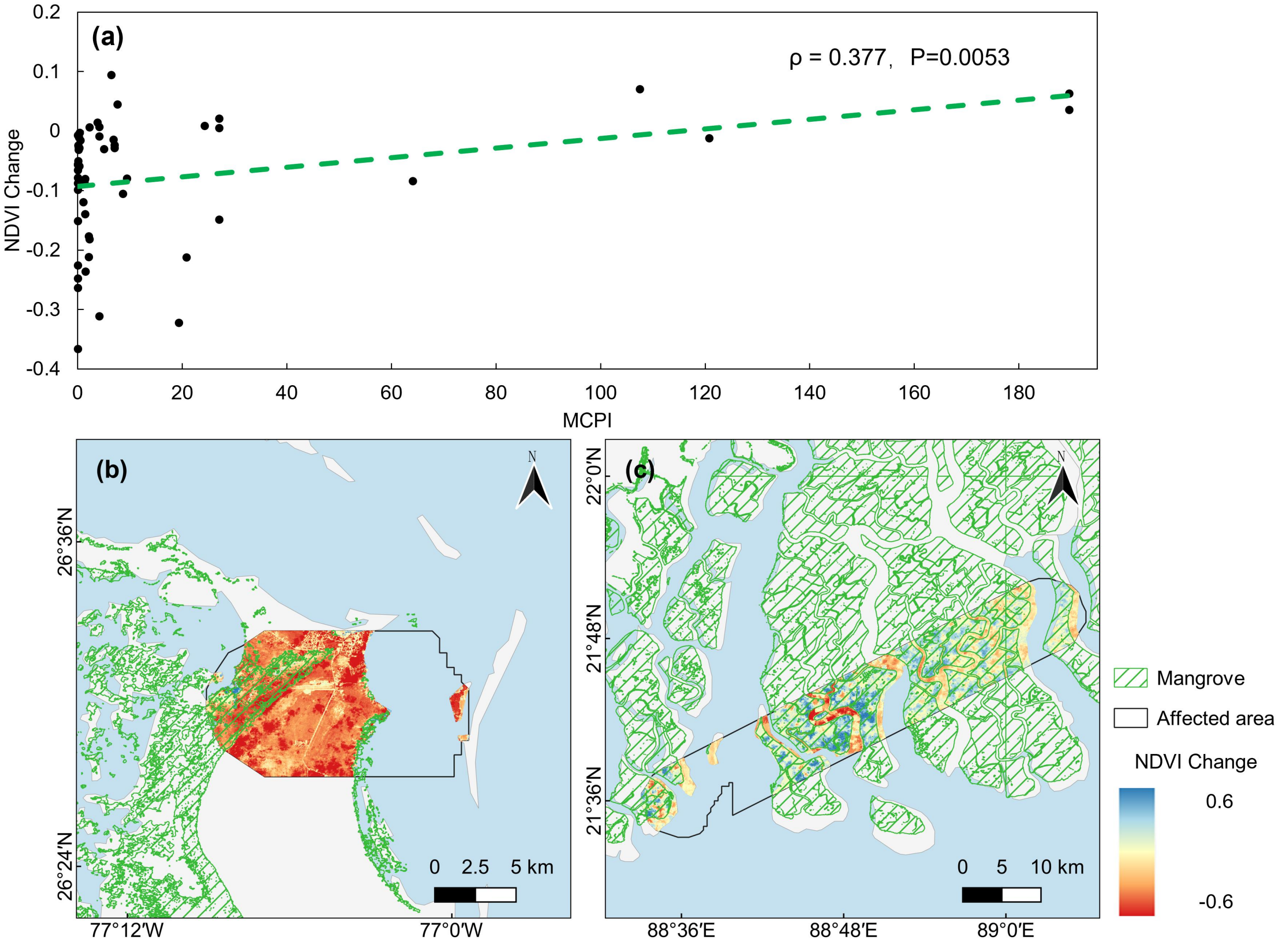

Supplementary Fig. 12 | Validation results for MCPI (Mangrove Coastal Protection Index). (a) depicts the relationship between the NDVI changes caused by storm events and mangrove MCPI, with a Spearman's rank-order correlation of 0.377 ($p = 0.0053$), (b) demonstrates a case of poor protection effectiveness in Great Abaco, with a significant decrease in NDVI (-0.366) under the influence of the storm event 'DORIAN', and (c) illustrates the Sundarbans mangrove forest in Bangladesh, where the NDVI did not significantly decrease after the storm 'BULBUL - MATMO' and even showed overall improvement (0.071), serving as an example of effective protection.

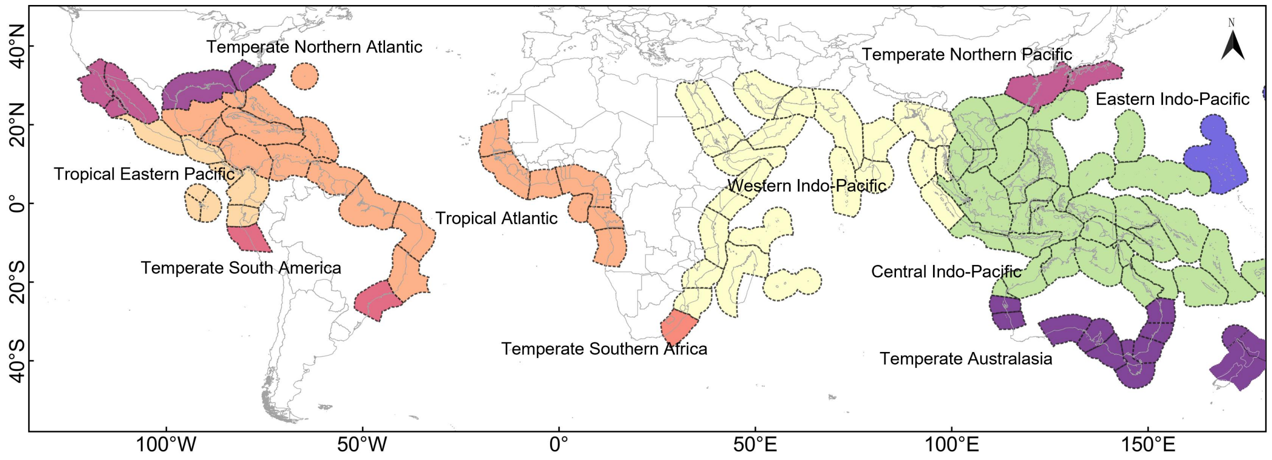

Supplementary Fig. 13 | Ecoregions within different realms relative to the study from Marine Ecoregions of the World (MEOW).

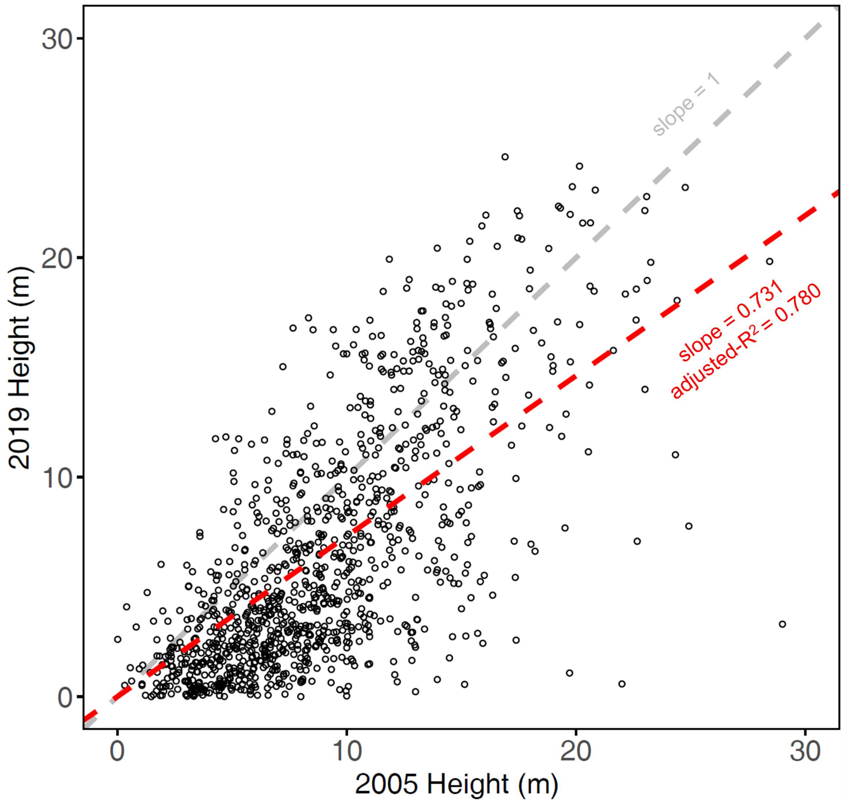

Supplementary Fig. 14 | Scatterplot of 2019 height against 2005 height. Dashed-line is 1:1 line, hence most 2019 heights are shorter; red dashed line is the fitted line for linear regression with the intercept term set to 0.

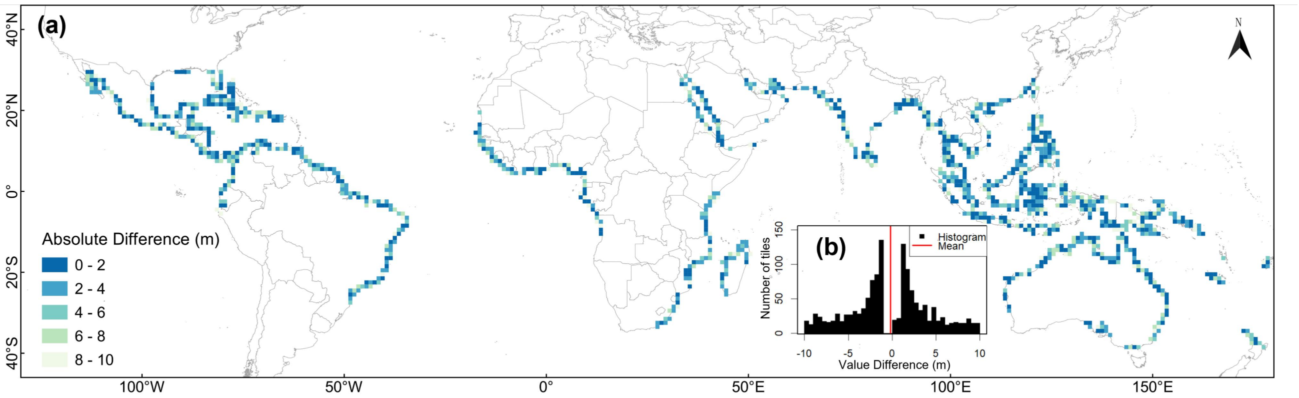

Supplementary Fig. 15 | Differences in Mangrove Width Extraction at 25m and 30m Resolutions. (a) depicts the spatial distribution of absolute differences in mangrove width extraction between the two resolutions, while (b) presents the histogram of value differences.

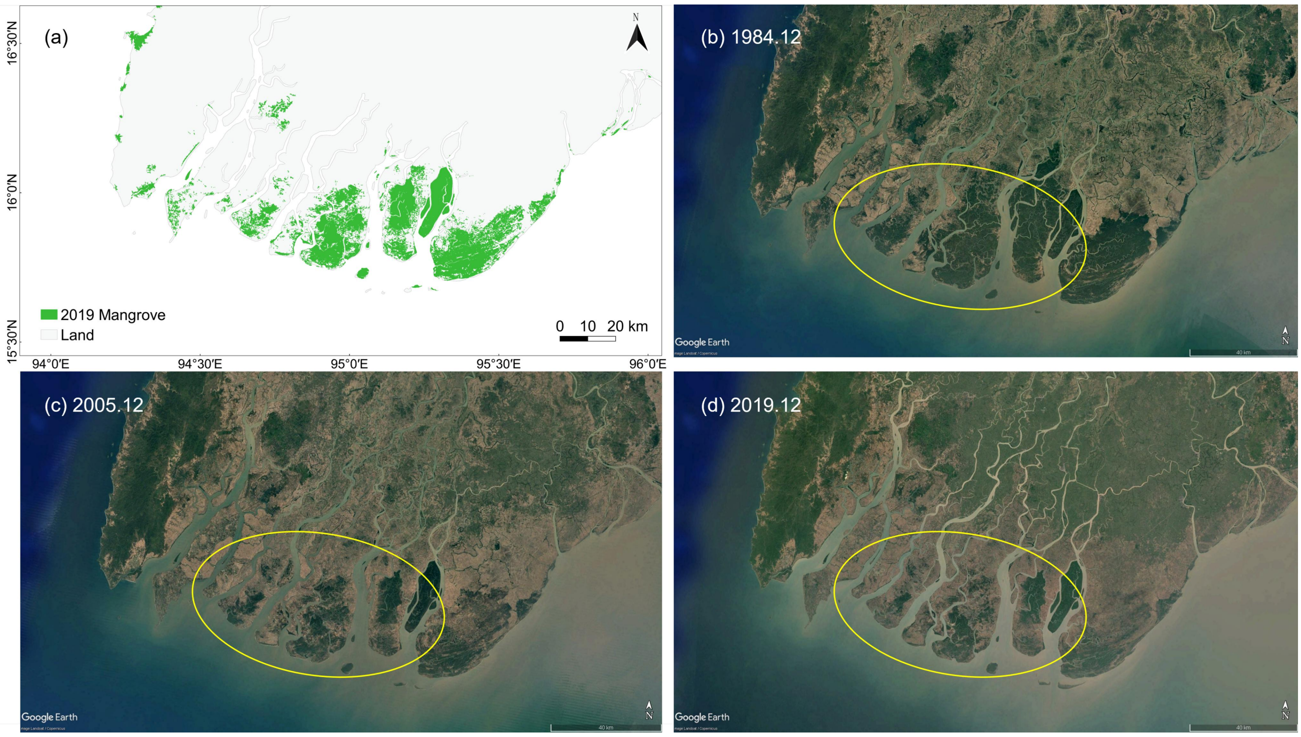

Supplementary Fig. 16 | Spot check of mangrove thinning for southwest Myanmar. (a) shows the distribution of mangroves in 2019 from GMW; (b), (c), and (d) are satellite images from December 1984, December 2005, and December 2019, respectively, obtained from Google Earth Pro. Over time, the mangrove areas have become increasingly fragmented, possibly due to sea level rise and flooding. Typical areas of change are highlighted by yellow ovals.

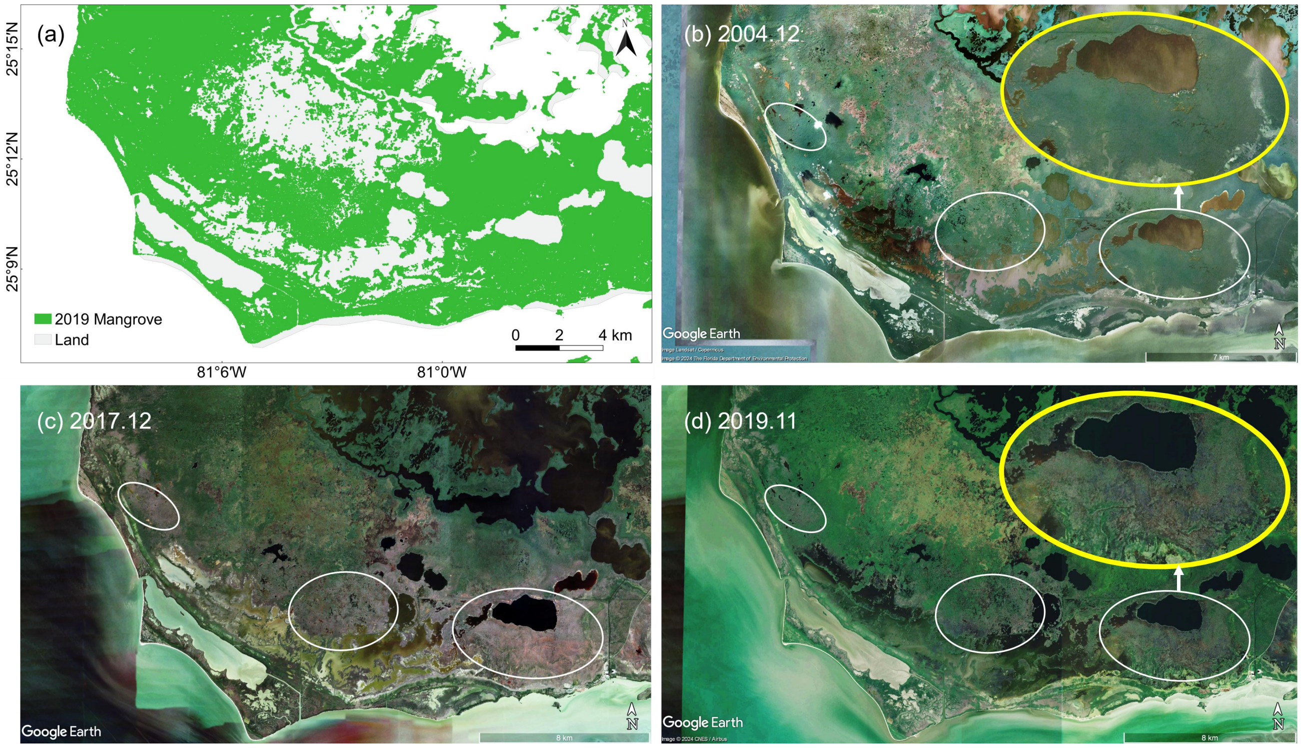

Supplementary Fig. 17 | Spot check for mangrove destruction for the southwestern corner of Florida, USA. (a) shows the distribution of mangroves in 2019; (b), (c), and (d) are satellite images from December 2004, December 2017, and November 2019, respectively, obtained from Google Earth Pro. The white ovals highlight areas where mangrove loss occurred following Hurricane Irma's strike in September 2017 and had not fully recovered by 2019. The yellow oval areas are an enlargement of the rightmost white oval area to facilitate viewing.

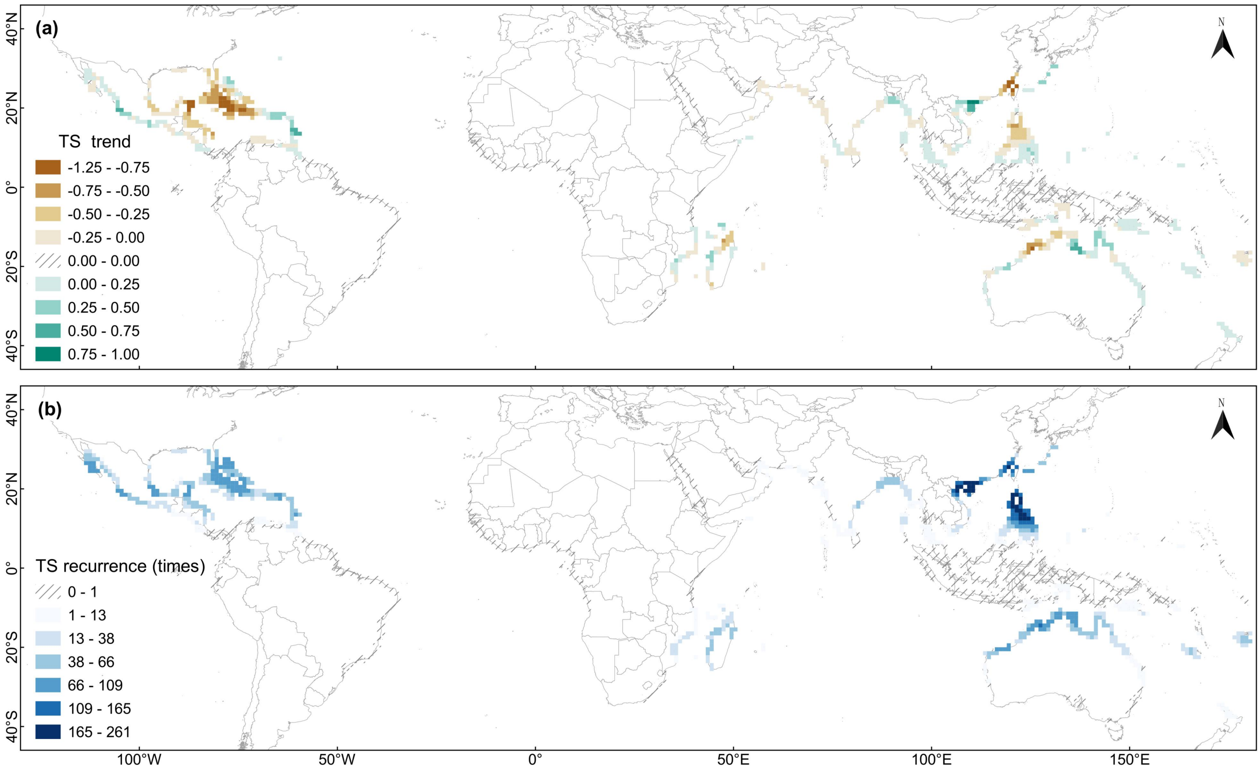

Supplementary Fig. 18 | The distribution of tropical cyclones trends (a) and recurrence (b). The tropical cyclones trends were derived through linear regression of the annual storm impact frequency, measured in times per year; the recurrence represents the total storm frequency from 2005 to 2019, measured in times. The dashed lines represent areas not affected by tropical storm events.

Supplementary Tables:

Supplementary Table 1 | List of Strong Tropical Cyclones Affecting Mangrove Hinterland Areas from 2017 to 2023.

ID	SID	Storm name	Time	MSWS	Tile	MCPI	NDVI Change
1	2019302N11118	BULBUL:MATMO	2019/11/10 0:00	70	N23E089	189.63455	0.036
2	2023274N09262	LIDIA	2023/10/11 0:00	120	N21W106	1.05360	-0.119
3	2022304N16287	LISA	2022/11/2 21:00	79	N18W089	5.00512	-0.030
4	2022304N16287	LISA	2022/11/2 18:00	75	N18W088	0.46207	-0.016
5	2019260N12262	LORENA	2019/9/19 3:00	67	N20W105	2.10143	-0.177
6	2019260N12262	LORENA	2019/9/19 6:00	65	N20W105	2.10143	-0.212
7	2020296N09137	MOLAVE	2020/10/25 15:00	72	N14E122	4.08708	-0.311
8	2020245N16285	NANA	2020/9/3 6:00	65	N17W089	8.60587	-0.105
9	2022311N21293	NICOLE	2022/11/9 23:00	65	N27W079	0.00013	-0.007
10	2022311N21293	NICOLE	2022/11/10 6:00	65	N28W081	6.82468	-0.014
11	2021236N12265	NORA	2021/8/28 18:00	75	N20W106	7.03885	-0.028
12	2021236N12265	NORA	2021/8/28 21:00	75	N20W106	7.03885	-0.023
13	2022272N14258	ORLENE	2022/10/3 15:00	66	N24W107	0.31392	-0.028
14	2022272N14258	ORLENE	2022/10/3 12:00	75	N23W107	0.15112	-0.032
15	2022272N14258	ORLENE	2022/10/3 14:35	70	N23W107	0.15112	-0.027
16	2021283N14258	PAMELA	2021/10/13 12:00	65	N24W107	0.31392	-0.059
17	2021346N05145	RAI	2021/12/16 15:00	115	N11E122	1.45479	-0.236
18	2021346N05145	RAI	2021/12/17 9:00	87	N11E118	120.73694	-0.012
19	2022293N15259	ROSLYN	2022/10/23 11:20	105	N22W106	9.40882	-0.079
20	2022293N15259	ROSLYN	2022/10/23 12:00	100	N23W106	1.26711	-0.086
21	2023236N20125	SAOLA	2023/9/2 3:00	72	N22E111	0.34349	-0.003
22	2021133N10071	TAUKTAE	2021/5/17 15:00	115	N21E071	0.00003	-0.055
23	2019074S08151	TREVOR	2019/3/23 0:00	105	S16E137	0.03738	-0.050
24	2020129N07134	VONGFONG	2020/5/14 3:00	97	N13E125	64.07578	-0.084
25	2020129N07134	VONGFONG	2020/5/14 15:00	75	N13E123	3.76813	0.014
26	2020129N07134	VONGFONG	2020/5/14 12:00	85	N13E124	20.79358	-0.212
27	2020129N07134	VONGFONG	2020/5/15 0:00	68	N14E122	4.08708	0.007
28	2020279N16284	DELTA	2020/10/7 9:00	90	N21W087	1.42554	-0.140
29	2020279N16284	DELTA	2020/10/7 10:30	90	N21W087	1.42554	-0.080
30	2019236N10314	DORIAN	2019/9/2 3:00	153	N27W078	0.00226	-0.225
31	2019236N10314	DORIAN	2019/9/2 9:00	140	N27W079	0.00013	-0.098
32	2019236N10314	DORIAN	2019/9/1 16:40	160	N27W078	0.00226	-0.366
33	2019236N10314	DORIAN	2019/9/1 18:00	160	N27W078	0.00226	-0.248
34	2022257N16312	FIONA	2022/9/18 19:20	75	N19W068	6.38088	0.095
35	2020276N17277	GAMMA	2020/10/3 16:45	65	N21W088	2.20633	-0.182
36	2020299N11144	GONI	2020/10/31 21:00	150	N14E124	19.28824	-0.322

37	2021225N15313	GRACE	2021/8/21 6:00	100	N21W098	24.26826	0.009
38	2021225N15313	GRACE	2021/8/19 9:45	75	N21W088	2.20633	0.007
39	2023296N18087	HAMOON	2023/10/24 18:00	74	N22E092	0.05111	-0.024
40	2022266N12294	IAN	2022/9/27 6:00	100	N22W084	4.07765	-0.009
41	2022266N12294	IAN	2022/9/27 8:30	110	N23W084	27.00474	0.021
42	2019302N11118	BULBUL:MATMO	2019/11/9 15:00	105	N22E088	7.60634	0.045
43	2021239N17281	IDA	2021/8/29 15:00	130	N30W091	0.02461	-0.066
44	2021239N17281	IDA	2021/8/27 23:25	70	N23W084	27.00474	0.005
45	2021239N17281	IDA	2021/8/28 0:00	70	N23W084	27.00474	-0.149
46	2021239N17281	IDA	2021/8/29 16:55	130	N30W091	0.02461	-0.078
47	2021239N17281	IDA	2021/8/29 18:00	125	N30W091	0.02461	-0.263
48	2019063S18038	IDAI	2019/3/14 21:00	100	S19E034	0.02608	-0.056
49	2019302N11118	BULBUL:MATMO	2019/11/9 18:00	95	N22E089	107.49846	0.071
50	2023099S11128	ILSA	2023/4/13 15:00	121	S19E119	0.21611	-0.011
51	2019302N11118	BULBUL:MATMO	2019/11/9 21:00	82	N23E089	189.63455	0.063
52	2020211N13306	ISAIAS	2020/8/1 15:00	66	N25W078	0.00000	-0.087
53	2020211N13306	ISAIAS	2020/8/1 13:00	70	N25W078	0.00000	-0.151
